# Some of Our Medical Universities and Colleges

**Published:** 1880-06

**Authors:** 


Some of our Medical Universities and Colleges.—It would be certain to draw largely upon our somewhat limited space, and therefore, cause the exclusion of other matter of more interest to our readers, were we to say all that could be justly said of several of our Southern and Western Medical Schools, and we therefore hope that should some that are worthy receive no notice at all, they will attribute our silence to the true cause, alluded to above. The University of Virginia has an old and well established medical department. The sessions are about nine months duration, and four professors teach during that period, the fundamental branches of medical science. The Richmond Medical College has also been in operation for many years, and has been quite successfully educating a number of excellent practitioners of the old dominion. The Medical College of South Carolina is an old, and for many years, quite a prominent school. Some of its graduates have been, and some are still, professors in their alma mater, and other schools. The late civil war exerted a disastrous effect upon this, as it did upon most of the medical schools of the South; but as long as the names of Dickson, Halbrook, Moultre, Geddings, Frost, Porcher, Michel and others, are mentioned as its professors, it must hold a prominent place in the mind of the profession.

The Medical Department of the University of Georgia, at Augusta, is another old and reputable school, and the profession will not be likely to forget the names of Antony, Eve, Dugas, Campbell, Doughty and others on the roll of distinguished professors.

The Savannah Medical College, at Savannah, Georgia, is a reputable Medical College, but like other schools of the South has experienced the ill-effects of the war. We had the honor of being its first Professor of Materia Medica and Therapeutics, a quarter of a century ago, and Arnold, Kollock, West and Howard, of our colleagues of those days, now rest in their graves, while their memories are cherished by a large circle of admiring friends. There are one or two schools in Atlanta, but they are summer schools, we believe. Mobile, New Orleans and Galveston, each have a medical school, but we are not apprized of their present condition or future prospects. We expect to revert to this subject again, in a future issue of the journal.

